# The Adulteration of Food

**Published:** 1901-03-02

**Authors:** 


### THE ADULTERATION OF FOOD.

We have received a long letter on the subject of glucose from a Liverpool manufacturer whose walk in life is the making of "boiled confectionery," including toffee, bottled sweets, wrapped sweets, and creams. He is a perfectly candid person, and, although he has not a word to say in favour of glucose, and indeed condemns it all round, he points out what we are afraid is strictly true in some branches of manufacturing industry, viz., that no such considerations are likely to stand in the way of its employment so long as it is cheaper than the sugar in place of which it is used. "In this branch of trade," he says, "profits are cut very fine, and therefore cheapness of the raw material is of the very first importance. As a manufacturer my point of view was a strictly commercial one, that is to say, that the quality of the goods as a foodstuff was immaterial. The selling quality was everything, and so long as the goods looked pretty, and were packed nicely into the boxes, we cared little enough how they tasted, and not a jot whether they were wholesome or unwholesome."

Needless to say under these circumstances they used glucose —not the sort that the brewers use, but the American kind. If too much of this stuff is used the goods produced are more susceptible to moisture than would otherwise be the case; but where the goods are sold wrapped in waxed paper—what are called "wrapped goods"—it appears that glucose can be employed to the extent of 30 per cent. or more. In making jam also glucose seems to be largely used, and "the peculiar thickness of factory-made jam as compared with the home-made article is due to its admixture." Now this candid manufacturer says many things against glucose. Considered as an element in confectionery it is without doubt, he says, "an outrageous fraud upon the public," for "its best friends can say no better of it than that it passes through the body unaltered in constitution, and thus does neither good nor harm"—a statement the exact accuracy of which we venture to doubt.

The chief charge, however, that he brings against it is that it is injurious to the teeth, and he suggests that much of the tendency to early decay of the teeth which is so commonly met with in the rising generation is due to the habit of eating glucose-containing sweets and jams. His point is that although it is possible in the making of glucose to free it from acid, this is not habitually done. Again, speaking "commercially," he says that it is better to have an excess of acid than to have the slightest excess of lime, for the latter would make the glucose appear milky, which would make it "unsaleable." Saleability is always the criterion. Hence he regards it as a fact that in practice the glucose used in confectionery contains free sulphuric acid, and as a consequential theory he holds that it is destructive to children's teeth.

Without going with him to the full extent of regarding this as the main cause of the prevalence of dental caries, it is easy to see that if it is true that the cheap "goodies" which are so largely consumed by children at the present day contain an appreciable quantity of free sulphuric acid they are likely to cause more injury than would arise from the mere eating of sugar; a cause which has been much discounted of late.

We are not, however, so much concerned with the suggestions made in our candid contributor's letter as with the illustration which it gives of the way in which the provision of food for the people is regarded by those who have to deal with the matter from a commercial point of view.

What is evident is that in commerce, whether it is a question of glucose, or beer, or toffee, or jam, in all alike it is not wholesomeness that is aimed at. Cheapness and "selling quality" are the great objects, and if the thing will sell and lead to business that is all that is asked for. If we recognise this we shall not be far from recognising what is the direction in which the Legislature can most usefully intervene for the protection of our food supply. It is but little use passing special laws prohibiting the use of this, that, or the other material in the making of certain specified articles of diet. We may be sure that the manufacturers will quickly find some other substitute still cheaper and perhaps still more injurious to take the place of the prohibited substance. What we have to do with is not the details of manufacture, which are liable to constant change and are incapable of being specified by Act of Parliament, but the condition of the finished product as supplied to the public. We want far greater strictness in regard to the analysis of everything which is sold for food, and far greater power to step in and at once prevent the sale of injurious or adulterated articles, than we now have.

At present the problem is fogged by questions of warranty and by the difficulty of apportioning the responsibility between the manufacturer, the wholesale dealer, and the retail tradesman, while overshadowing the whole subject, and rendering it almost impossible to make any headway against vested interests, is the stolid and stupid indifference which possesses the public in regard to its food supply; an indifference only varied by an equally stupid scare whenever a flagrant case of food-poisoning is brought to light, and an even more stupid demand for remedies directed solely to the particular case which has roused it to a momentary sense of danger.

